# Supplementary material for: Application of artificially intelligent systems for the identification of discrete fossiliferous levels
Source: PeerJ. 2020 Mar 11;8:e8767. doi: 10.7717/peerj.8767 (PMC7071820; doi:10.7717/peerj.8767)
Supplement: Supplemental Information 3 [file peerj-08-8767-s003.docx]

|  | | **Bat10 - y slice (RF/SVM)** | | |  | | **Bat10 - x slice (RF/SVM)** | | |
| --- | --- | --- | --- | --- | --- | --- | --- | --- | --- |
|  |  | Reference Label | | |  |  | Reference Label | | |
|  |  | I | II | III |  |  | I | II | III |
| Predicted label | I | 159/159 | 0/0 | 0/0 | Predicted label | I | 207/209 | 0/0 | 0/0 |
|  | II | 0/0 | 113/113 | 2/0 |  | II | 1/1 | 87/87 | 0/0 |
|  | III | 0/0 | 0/0 | 116/118 |  | III | 1/1 | 0/0 | 85/85 |
|  | | **Bat3 - left slice (RF/SVM)** | | |  | | **Bat3 - right slice (RF/SVM)** | | |
|  |  | Reference Label | | |  |  | Reference Label | | |
|  |  | I | II | III |  |  | I | II | III |
| Predicted label | I | 48/48 | 0/0 | 0/0 | Predicted label | I | 16/16 | 0/0 | 0/0 |
|  | II | 0/0 | 134/139 | 0/1 |  | II | 0/0 | 37/37 | 2/0 |
|  | III | 0/0 | 5/0 | 120/119 |  | III | 0/0 | 0/0 | 42/44 |
